# *Phycobium rhodophyticola* gen. nov., sp. nov. and *Aliiphycobium algicola* gen. nov., sp. nov., isolated from the phycosphere of marine red algae

**DOI:** 10.71150/jm.2503014

**Published:** 2025-06-30

**Authors:** Jeong Min Kim, Woonhee Baek, Byeong Jun Choi, Hülya Bayburt, Jae Kyeong Lee, Sung Chul Lee, Che Ok Jeon

**Affiliations:** Department of Life Science, Chung-Ang University, Seoul 06974, Republic of Korea

**Keywords:** *Phycobium rhodophyticola*, *Aliiphycobium algicola*, marine red algae, new taxa (*Pseudomonadota*), metabolic interaction, phycosphere

## Abstract

Two Gram-stain-negative, strictly aerobic, non-motile, rod-shaped bacteria, designated D3-12ᵀ and G2-2ᵀ, were isolated from the phycosphere of marine red algae. Both strains exhibited catalase- and oxidase-positive activities. Strain D3-12ᵀ grew optimally at 30°C, pH 7.0, and 2.0–3.0% (w/v) NaCl, while strain G2-2ᵀ showed optimal growth at 30°C, pH 7.0, and 2.0% NaCl. Ubiquinone-10 was the sole respiratory quinone in both strains. The major fatty acids (> 5%) in strain D3-12ᵀ were feature 8 (C_18:1_ *ω*7*c* and/or C_18:1_ *ω*6*c*), 11-methyl-C_18:1_ *ω*7*c*, and C_16:0_, while strain G2-2ᵀ contained summed feature 8 and C_16:0_. The predominant polar lipids in strain D3-12ᵀ were phosphatidylcholine, phosphatidylglycerol, and phosphatidylethanolamine, whereas strain G2-2ᵀ contained phosphatidylglycerol and diphosphatidylglycerol. The genomic DNA G + C content was 59.9% for strain D3-12ᵀ and 60.2% for strain G2-2ᵀ. Phylogenetic analyses based on 16S rRNA and whole-genome sequences placed both strains into distinct lineages within the family *Roseobacteraceae*, separate from previously described genera. Genome-based relatedness metrics, including average nucleotide identity, digital DNA-DNA hybridization, average amino acid identity, and percentage of conserved proteins, further confirmed that these strains represent novel genera. Based on phenotypic, chemotaxonomic, and molecular characteristics, strains D3-12ᵀ and G2-2ᵀ are proposed as novel genera: *Phycobium rhodophyticola* gen. nov., sp. nov. (D3-12ᵀ = KACC 22712ᵀ = JCM 35528ᵀ) and *Aliiphycobium algicola* gen. nov., sp. nov. (G2-2ᵀ = KACC 22602ᵀ = JCM 35752ᵀ). Additionally, metabolic features relevant to interactions with marine algae, including genes associated with carbohydrate-active enzymes, vitamin biosynthesis, phenylacetic acid production, and bacterioferritin synthesis, were bioinformatically investigated.

## Introduction

The family *Roseobacteraceae* within the phylum *Pseudomonadota* was recently established based on whole-genome phylogenetic and genotypic analyses of roseobacter clade members, which were originally classified as a broad group of marine bacteria within the family *Rhodobacteraceae* (Liang et al., 2021). As of May 2025, *Roseobacteraceae* comprises 137 validly published genera (https://lpsn.dsmz.de/family/roseobacteraceae) exhibiting diverse phenotypic, physiological, and ecological traits, which may contribute to marine biogeochemical cycles, including sulfur and carbon cycling, aerobic anoxygenic photosynthesis, and carbon monoxide oxidation, and symbiotic interactions with both micro- and macro-organisms in marine environments (Ding et al., 2023; Geng and Belas, 2010). *Roseobacteraceae* strains have been isolated from various marine environments, including seawater (Hwang and Cho, 2008), tidal mudflats (Lee et al., 2020), sea sand (Thongphrom et al., 2017), marine solar saltern (Wang et al., 2023), biofilms (Cui et al., 2025), and polar sea ice (Gosink et al., 1997). Notably, many *Roseobacteraceae* members have been associated with marine algae, including dinoflagellates (Yang et al., 2018), diatoms (Crenn et al., 2016), microalgae (Jung et al., 2019), and other macroalgae (Han et al., 2024; Lee et al., 2024a).

The marine coastal regions are known for their high biodiversity, including fish, macroalgae, diatoms, and microorganisms (Tak et al., 2024; Yang et al., 2024). In particular, macroalgae serve as crucial habitats for various marine organisms, including fish and microorganisms (Evans et al., 2014; Lee et al., 2024b), and are also commercially important as food sources (Wells et al., 2017). Bacteria in the phycosphere play essential roles in algal growth and development. Marine red algae (phylum Rhodophyta) represent one of the oldest and most diverse groups of marine algae, comprising over 7,000 recognized species and contributing significantly to primary production (Guiry, 2024), leading to increasing research on metabolic interactions between bacteria and marine red algae (Cirri and Pohnert, 2019; Kim et al., 2024b). As part of our ongoing research on these interactions, we have isolated numerous novel bacterial strains from the phycosphere of marine red macroalgae (Bayburt et al., 2024; Jin et al., 2023; Kim et al., 2024a; Lee et al., 2024c). In this study, we also isolated two putative novel genus strains within the family *Roseobacteraceae* from marine red macroalgae and characterized their taxonomy using a polyphasic approach.

## Materials and Methods

### Isolation of bacterial strains

Strains D3-12ᵀ and G2-2ᵀ were isolated from the phycosphere of the marine red algae *Melanothamnus japonicus* and *Chondrus* sp., respectively, collected from the coastal regions of Daejin (38°30′14″N, 128°25′36″E) and Gonghyeonjin (38°21′21″N, 128°30′45″E) in Gangwon Province, Republic of Korea, as previously described (Kim et al., 2024a). Briefly, the collected algal samples were thoroughly washed with artificial seawater (ASW; 20.0 g NaCl, 2.9 g MgSO_4_, 4.5 g MgCl_2_·6H_2_O, 0.6 g KCl, and 1.8 g CaCl_2_·2H_2_O per liter) using mechanical vortexing to remove loosely attached microorganisms. The algae were then homogenized using a mechanical homogenizer and serially diluted in ASW. Aliquots of 100 μl from each dilution were spread onto marine agar (MA; MBcell, Korea) and incubated aerobically at 25°C for 7 days. Colonies grown on MA were screened by PCR amplification of the 16S rRNA gene using universal primers 27F (5′-AGA GTT TGA TCM TGG CTC AG-3′) and 1492R (5′-TAC GGY TAC CTT GTT ACG ACT T-3′) (Lane, 1991). PCR products were digested with the restriction enzymes HaeIII and HhaI, and the resulting fragment patterns were analyzed on a 2% agarose gel. Representative amplicons showing unique or identical fragment patterns were partially sequenced using the universal primer 340F (5′-CCT ACG GGA GGC AGC AG-3′). The obtained sequences were compared with those of validly published type strains using the EzBioCloud server (https://www.ezbiocloud.net/identify) (Yoon et al., 2017). Two putative novel strains, D3-12ᵀ and G2-2ᵀ, were selected for further taxonomic analysis. These strains were routinely cultured on MA at 30°C for 2 days and preserved at -80°C in marine broth (MB; MBcell) supplemented with 15% (v/v) glycerol.

### Phylogenetic analysis of 16S rRNA gene sequences and ecological distribution assessment

The 16S rRNA gene amplicons of strains D3-12ᵀ and G2-2ᵀ, initially amplified using primers 27F and 1492R, were further sequenced with universal primers 518R (5′-ATT ACC GCG GCT GCT GG-3′) and 805F (5′-GAT TAG ATA CCC TGG TAG TC-3′) (Kim et al., 2024a). Sequences obtained from primers 340F, 518R, and 805F were assembled to generate nearly complete 16S rRNA gene sequences. Sequence similarities of the 16S rRNA genes of strains D3-12ᵀ and G2-2ᵀ were compared with those of validly published type strains using the nucleotide similarity search tool on the EzBioCloud server. The 16S rRNA gene sequences of strains D3-12ᵀ and G2-2ᵀ, along with those of closely related type strains, were aligned, and phylogenetic trees were constructed using the neighbor-joining (NJ), maximum-likelihood (ML), and maximum-parsimony (MP) methods in MEGA11 software (Tamura et al., 2021), with bootstrap values calculated from 1,000 replications to assess the robustness of the phylogenetic relationships. The Kimura two-parameter model, the nearest-neighbour-interchange heuristic search method, and the pairwise deletion options were used in constructing the NJ, MP, and ML trees, respectively. Based on the 16S rRNA gene sequence similarities, *Rhodalgimonas zhirmunskyi* KCTC 72611^T^, *Cognatishimia maritima* KCTC 23347ᵀ, *Marimonas lutisalis* KCTC 62376ᵀ, *Aquicoccus porphyridii* KACC 18806ᵀ, *Marimonas arenosa* KCTC 52189ᵀ, and *Ponticoccus litoralis* KCCM 90028ᵀ were selected as reference strains for comparative analyses of genomic features, fatty acid composition, and phenotypic characteristics.

The potential ecological distribution of strains D3-12ᵀ and G2-2ᵀ was evaluated by comparing their 16S rRNA gene sequences with metagenomic 16S rRNA amplicon datasets from diverse environments—including seawater, freshwater, marine and freshwater sediments, oysters, corals, soil, plants, air, epibionts, and gut microbiomes—using the Integrated Microbial Next-Generation Sequencing (IMNGS) platform (Lagkouvardos et al., 2016), with a sequence similarity threshold of 99.0%.

### Whole-genome sequencing, phylogenomic analysis, and assessment of genome-relatedness

For whole-genome sequencing of strains D3-12ᵀ, G2-2ᵀ, and *P. litoralis* KCCM 90028ᵀ, genomic DNA was extracted from cells cultured in MB using the Wizard Genomic DNA Purification Kit (Promega, USA), following the manufacturer’s protocol. The extracted genomic DNA was sequenced using the Oxford Nanopore MinION platform (ONT, UK), and the resulting sequencing reads were de novo assembled using Unicycler (version 0.4.9; Wick et al., 2017) for strain D3-12ᵀ and Flye (version 2.9.1; Kolmogorov et al., 2019) for strains G2-2ᵀ and *P. litoralis* KCCM 90028ᵀ. Genome completeness and contamination were assessed using CheckM2 (version 1.0.2; Chklovski et al., 2023). Phylogenomic analysis of strains D3-12ᵀ and G2-2ᵀ, along with closely related type strains, was conducted using the Genome Taxonomy Database Toolkit (GTDB-Tk) based on the concatenated protein sequences of 120 single-copy marker genes (bac120 marker set) (Chaumeil et al., 2020). Sequence alignment and construction of an ML phylogenomic tree were performed in MEGA11, with bootstrap values calculated from 1,000 replications.

Average nucleotide identity (ANI) and digital DNA-DNA hybridization (dDDH) values between strains D3-12ᵀ, G2-2ᵀ, and their closest type strains were calculated using the Orthologous ANI Tool (OAT, version 0.93.1; www.ezbiocloud.net/tools/orthoani) (Lee et al., 2016) and the Genome-to-Genome Distance Calculator (GGDC 3.0; https://ggdc.dsmz.de/ggdc.php) using formula 2 (Meier-Kolthoff et al., 2013), respectively. Average amino acid identity (AAI) and percentage of conserved proteins (POCP) were calculated using EzAAI (version 1.2.3; Kim et al., 2021) and the method described by Qin et al. (2014), respectively.

### Genomic characterization and bioinformatic analysis of algae-associated genes

The genome sequences of strains D3-12ᵀ and G2-2ᵀ and *P. litoralis* KCCM 90028ᵀ were submitted to GenBank and annotated using the NCBI Prokaryotic Genome Annotation Pipeline (Tatusova et al., 2016). Carbohydrate-Active Enzyme (CAZyme) genes in the genomes of strains D3-12ᵀ and G2-2ᵀ, as well as in closely related *Roseobacteraceae* species, were identified using the dbCAN3 meta server (https://bcb.unl.edu/dbCAN2/blast.php) with the CAZyme database as a reference (Zheng et al., 2023). In addition, the presence of metabolic genes potentially involved in symbiotic interactions with marine algae was assessed using BLASTP, by comparing reference protein sequences from the UniProt database (https://www.uniprot.org) against the genomic datasets.

### Phenotypic and biochemical characterization

The growth of strains D3-12^T^ and G2-2^T^ was assessed on various bacteriological agar media, including MA, Reasoner’s 2A (R2A) agar, Luria-Bertani (LB) agar, tryptic soy agar (TSA), and nutrient agar (NA) (all from MBcell), each supplemented with ~2% NaCl. Cultures were incubated at 30°C for 2 days. Growth was also assessed on MA across a temperature range of 5°C to 40°C (in 5°C increments) and in MB adjusted to pH values from 4.0 to 10.0 (in 1.0 unit intervals), incubated at 30°C for 2 days. pH adjustments were made using sodium citrate (pH 4.0–5.0), sodium phosphate (pH 6.0–8.0), and sodium carbonate (pH 9.0–10.0) buffer systems, with final corrections performed after autoclaving if necessary. Salt tolerance was tested for 2 days in MB media prepared in the laboratory with varying NaCl concentrations (0% to 10% in 1% increments), following the standard MB composition. Cell morphology and motility were observed following incubation on MA at 30°C for 2 days using phase-contrast microscopy (Zeiss Axio Scope.A1; Carl Zeiss, Germany). For transmission electron microscopy (TEM), cells were placed on Formvar-coated copper grids (Electron Microscopy Sciences, USA), negatively stained with 2% (w/v) uranyl acetate for 15 s, and examined using a JEM-1010 TEM (JEOL, Japan).

Gram staining was performed using a commercial kit (bioMérieux, France) according to the manufacturer’s instructions. Catalase activity was determined by observing oxygen bubble formation in a 3% (v/v) hydrogen peroxide solution (Junsei, Japan), while oxidase activity was assessed by the oxidation of 1% (w/v) tetramethyl-*p*-phenylenediamine (Merck, USA), following the method described by Smibert and Krieg (1994). Anaerobic growth was evaluated on MA under anaerobic conditions using the GasPak Plus system (BBL, USA) at 30°C for 3 weeks. Phenotypic characteristics of strains D3-12ᵀ and G2-2ᵀ were compared with those of five reference strains under identical conditions at their respective optimal growth temperatures. The ability to hydrolyze esculin, casein, starch, tyrosine, Tween 20, and Tween 80 was tested on MA as previously described by Lányi (1987). Additional biochemical traits were assessed using the API 20NE system (bioMérieux), following the manufacturer’s instructions, with inocula prepared by suspending cells in ASW.

### Chemotaxonomic characterization

To analyze respiratory isoprenoid quinones, strains D3-12^T^ and G2-2^T^ were cultivated to their exponential growth phases in MB at 30°C. Bacterial cells were harvested by centrifugation, and their respiratory isoprenoid quinones were extracted, as previously described (Minnikin et al., 1984). The extracted quinones were analyzed using a high-performance liquid chromatography system (LC-20A; Shimadzu, Japan) equipped with a diode array detector (SPD-M20A; Shimadzu) and a reversed-phase column (250 × 4.6 mm, Kromasil; Akzo Nobel). Methanol-isopropanol (2:1, v/v) was used as the eluent at a flow rate of 1 ml/min. For cellular fatty acid analysis, strains D3-12^T^ and G2-2^T^, along with reference strains, were grown in MB at their optimal temperatures, and their bacterial cells were harvested at the same growth stage (exponential phase; optical density, OD_600_ = 0.7–0.8). The fatty acids from the microbial cells were saponified, methylated, and extracted using the standard MIDI protocol, then analyzed by gas chromatography (Hewlett Packard 6890). Cellular fatty acids were identified and quantified using the RTSBA6 database of the Microbial Identification System (Sherlock ver. 6.0B) (Sasser, 1990). Polar lipids of strains D3-12^T^ and G2-2^T^ were analyzed using two-dimensional thin-layer chromatography, with cells harvested during the exponential growth phase, following the method described by Minnikin et al. (1977). Different reagents were used to detect various polar lipids, including 10% ethanolic molybdophosphoric acid (for total polar lipids), ninhydrin (for aminolipids), Dittmer-Lester (for phospholipids), *α*-naphthol/sulfuric acid (for glycolipids), and Dragendorff (for phosphatidylcholine; PC) reagents. The presence or absence of PC, phosphatidylglycerol (PG), phosphatidylethanolamine (PE), and diphosphatidylglycerol (DPG) in strains D3-12^T^ and G2-2^T^ were confirmed using standard polar lipid compounds from Sigma-Aldrich (USA).

## Results and Discussion

### Phylogenetic characteristics of strains D3-12^T^ and G2-2^T^ based on 16S rRNA gene sequences

**Fig. 1.**
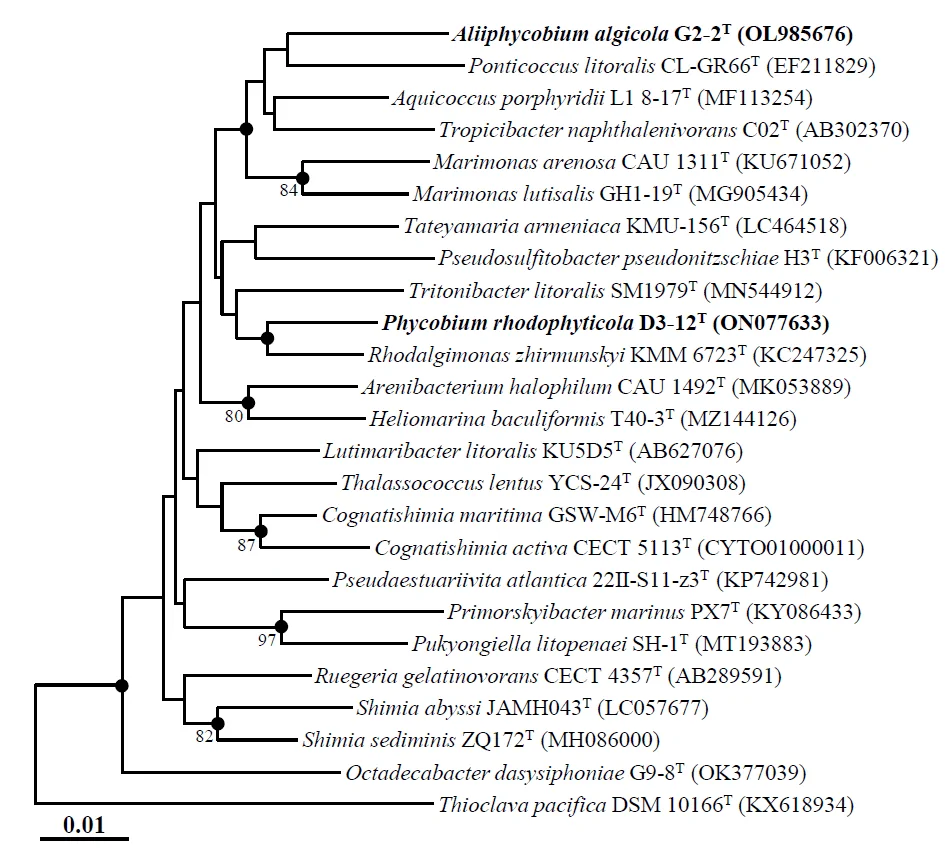
Neighbor-joining tree showing the phylogenetic relationships between strains D3-12^T^ and G2-2^T^ and their closely related type strains, based on 16S rRNA gene sequences. Numbers at the nodes represent bootstrap percentages from 1,000 replicates; only values greater than 70% are shown. Filled circles (●) mark nodes that were also supported in the maximum-likelihood and maximum-parsimony trees. *Thioclava pacifica* DSM 10166^T^ (KX618934) was employed as the outgroup. Scale bar represents 0.01 nucleotide substitutions per site.

The putative novel genus strains D3-12ᵀ and G2-2ᵀ, members of the family *Roseobacteraceae* isolated from the phycosphere of marine algae, yielded nearly complete 16S rRNA gene sequences (1,406 bp for D3-12ᵀ and 1,401 bp for G2-2ᵀ) through sequencing and assembly of 16S rRNA gene amplicons using primers 340F, 518R, and 805F. The 16S rRNA gene sequence similarity between strains D3-12ᵀ and G2-2ᵀ was 95.6%, which is below the commonly accepted threshold of 98.5–98.7% for species delineation based on 16S rRNA gene sequences (Riesco and Trujillo, 2024), indicating that the two strains are likely distinct. Comparative sequence analysis showed that strain D3-12ᵀ was most closely related to *R. zhirmunskyi* KMM 6723^T^, *C. maritima* GSW-W6^T^, and *M. lutisalis* GH1-19^T^, with sequence similarities of 97.8%, 96.8%, and 96.5%, respectively. In contrast, strain G2-2ᵀ was most closely related to *A. porphyridii* L1 8-17^T^, *M. arenosa* CAU 1311^T^, and *P. litoralis* KCCM 90028^T^, with sequence similarities of 96.7%, 96.4%, and 96.2%, respectively. Phylogenetic analysis using the NJ algorithm showed that both strains formed distinct lineages separate from other genera within the family *Roseobacteraceae*, with low bootstrap values (Fig. 1). Phylogenetic trees generated using the ML and MP algorithms further confirmed the distinct placement of strains D3-12ᵀ and G2-2ᵀ from other genera within the family *Roseobacteraceae* (Fig. S1). Together, these comparative and phylogenetic analyses based on 16S rRNA gene sequences strongly suggest that strains D3-12ᵀ and G2-2ᵀ likely represent two novel genera within the family *Roseobacteraceae*.

### Ecological insights of strains D3-12^T^ and G2-2^T^ based on their distribution

Ecological habitat distribution analysis of strains D3-12ᵀ and G2-2ᵀ using the IMNGS platform revealed that their 16S rRNA gene sequences were detected in metagenomic datasets from a wide range of environments (Table S1). Notably, their sequences were abundantly found in datasets associated with marine environments, including *Panulirus ornatus*, marine and seawater metagenomes, *Seminavis robusta*, marine sediment metagenomes, and algae, suggesting that marine ecosystems likely represent their primary ecological habitats. Interestingly, the 16S rRNA gene sequences of D3-12ᵀ and G2-2ᵀ showed differing distribution patterns across various environments, implying that although both strains were isolated from the phycosphere of marine macroalgae, they may occupy distinct ecological niches. Additionally, their sequences were also identified—though at lower abundance—in non-marine metagenomic datasets, including those from terrestrial, compost, plant, and freshwater environments, indicating a potentially broad ecological distribution, although strains D3-12ᵀ and G2-2ᵀ, as well as their closest known relatives, have been exclusively isolated from marine environments (Feng et al., 2018; Hwang and Cho, 2008; Lee et al., 2020; Park et al., 2012; Thongphrom et al., 2017).

### Whole genome sequencing, phylogeny based on genome sequences, and genome relatedness

De novo assembly of MinION sequencing reads yielded complete genomes for strains D3-12^T^ and G2-2^T^, with genome sizes of approximately 4,496 kb and 3,786 kb, and average genome coverages of 42.2× and 162.0×, respectively. In contrast, assembly for *P. litoralis* KCCM 90028^T^ resulted in a draft genome of 4,790 kb, comprising five contigs, with an average coverage of 22.0× and an N50 value of 3,898 kb. The 16S rRNA gene sequences identified in the assembled genomes were consistent with those obtained via PCR-based sequencing. Genome quality assessment using CheckM2 indicated completeness values of 98.9%, 99.2%, and 91.1%, and contamination rates of 0.8%, 1.3%, and 1.0% for strains D3-12ᵀ, G2-2ᵀ, and *P. litoralis* KCCM 90028ᵀ, respectively, confirming that all genomes meet the criteria for high-quality genome assemblies (completeness ≥ 90%, contamination ≤ 10%) (Chklovski et al., 2023).

**Fig. 2.**
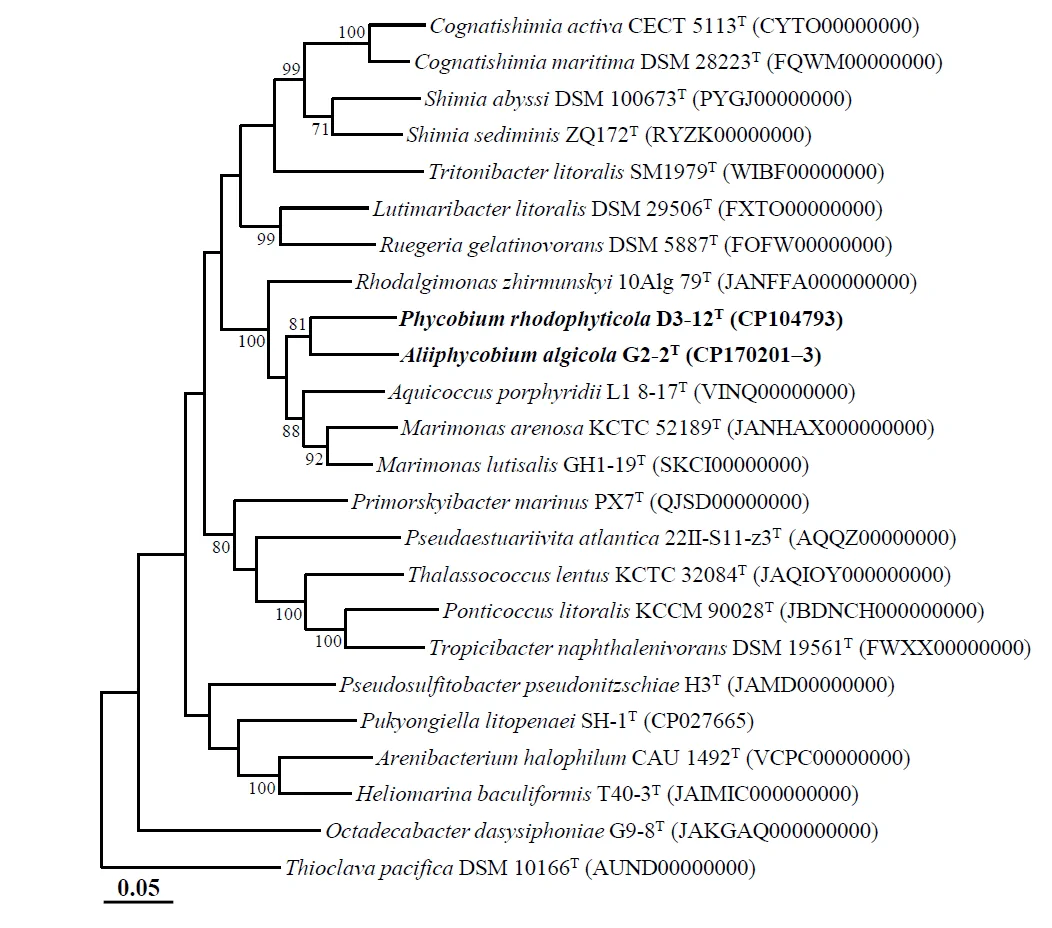
Phylogenomic tree showing the phylogenetic relationships between strains D3-12^T^ and G2-2^T^ and their closely related type strains, based on the concatenated protein sequences of 120 ubiquitous single-copy marker genes (bac120 marker set) of GTDB-Tk. Numbers at the nodes represent bootstrap percentages from 1,000 replicates; only values greater than 70% are shown. *Thioclava pacifica* DSM 10166^T^ (AUND00000000) was employed as the outgroup. Scale bar represents 0.05 substitutions per amino acid.

Phylogenomic analysis based on the concatenated protein sequences of 120 single-copy marker genes revealed that strains D3-12ᵀ and G2-2ᵀ clustered together within the family *Roseobacteraceae* (Fig. 2), in contrast to 16S rRNA gene sequence-based phylogenetic analyses, which placed them in distinct lineages (Figs. 1 and S1). Despite their phylogenomic clustering, the two strains exhibited low genome relatedness, with ANI and dDDH values of 74.5% and 18.7%, respectively (Table S2), and AAI and POCP values of 75.0% and 61.3%, respectively (Table S3). Although a genus-level POCP threshold of 50% was initially proposed for prokaryotes (Qin et al., 2014), several exceptions within the roseobacter group suggest that POCP alone may not reliably delineate genera in this group (Wirth and Whitman, 2018). Therefore, it has been suggested that POCP should be used in conjunction with other genomic indices, such as AAI, for robust genus classification. Luo et al. (2014) noted that AAI values between members of distinct genera typically range from 60% to 80%, rarely exceeding 85%. Therefore, despite clustering together in the phylogenomic tree, strains D3-12ᵀ and G2-2ᵀ represent distinct genera.

Moreover, phylogenomic analysis showed that both strains formed phylogenetic lineages clearly separated from other genera within the family *Roseobacteraceae*. Comparative genomic analyses with closely related genera further confirmed their distinctiveness, with ANI, dDDH, AAI, and POCP values of ≤ 75.5%, ≤ 21.0%, ≤ 76.6%, and ≤ 68.3%, respectively (Tables S2 and S3). Taken together, these results strongly support the classification of strains D3-12ᵀ and G2-2ᵀ as representatives of two distinct novel genera within the family *Roseobacteraceae*.

### Genomic features and functional genes related to algal interactions

The complete genome of strain D3-12^T^ consists of a single circular chromosome (4,496 kb with a G + C content of 59.9%) and encodes 4,393 genes, including 4,049 protein-coding sequences, one rRNA operon (16S, 23S, and 5S), 42 tRNA genes, and three noncoding RNA genes. Strain G2-2^T^ has a complete genome with one circular chromosome (3,565 kb with a G + C content of 60.5%) and two plasmids (217.6 kb with 57.2% G + C content and 3.5 kb with 43.4% G + C content), encoding a total of 3,681 genes, including 3,393 protein-coding sequences, one rRNA operon (16S, 23S, and 5S), 42 tRNA genes, and three noncoding RNA genes. The draft genome of *P. litoralis* KCCM 90028^T^, consisting of five contigs (4,790 kb with a G + C content of 67.3%), contains 4,772 genes, including 3,931 protein-coding sequences, three rRNA operons (16S, 23S, and 5S), 47 tRNA genes, and three noncoding RNA genes. The G + C contents of strains D3-12^T^ and G2-2^T^, based on their entire genomes, were 59.9% and 60.2%, respectively. The general genomic features of these strains, along with closely related *Roseobacteraceae* species, are summarized in Table 1 and show similarities to other members of the *Roseobacteraceae* family.

Algae are primarily composed of polysaccharides, which constitute key components of their extracellular matrices, cell walls, and storage materials. Consequently, the ability to degrade diverse algal polysaccharides is a critical trait of heterotrophic bacteria associated with marine algae (Mühlenbruch et al., 2018). Strain D3-12ᵀ encodes 52 CAZymes, including 37 glycosyltransferases (GTs)—a higher number than observed in strain G2-2ᵀ and other closely related strains (Table S4)—suggesting a broader capacity for polysaccharide utilization. Notably, the GT1 family, which encodes UDP-glucuronosyltransferases involved in the glycosylation of sugars and secondary metabolites (Ulvskov et al., 2013), was uniquely detected in strain D3-12ᵀ but not in strain G2-2ᵀ or any related strains. The presence of GT1 may indicate that strain D3-12ᵀ has the potential to modify algal-derived compounds, such as sulfated polysaccharides and polyphenols, thereby possibly enhancing their bioavailability and promoting metabolic interactions in the algal phycosphere. Additionally, carbohydrate esterase (CE) genes—responsible for removing ester-linked modifications during the initial stages of polysaccharide degradation (Li et al., 2022)—were present in D3-12ᵀ but absent in strain G2-2ᵀ (Table S4). This absence might suggest a reduced capacity for independent polysaccharide degradation in strain G2-2ᵀ, although alternative degradation mechanisms or synergistic interactions with other phycosphere bacteria cannot be excluded.

**Fig. 3.**
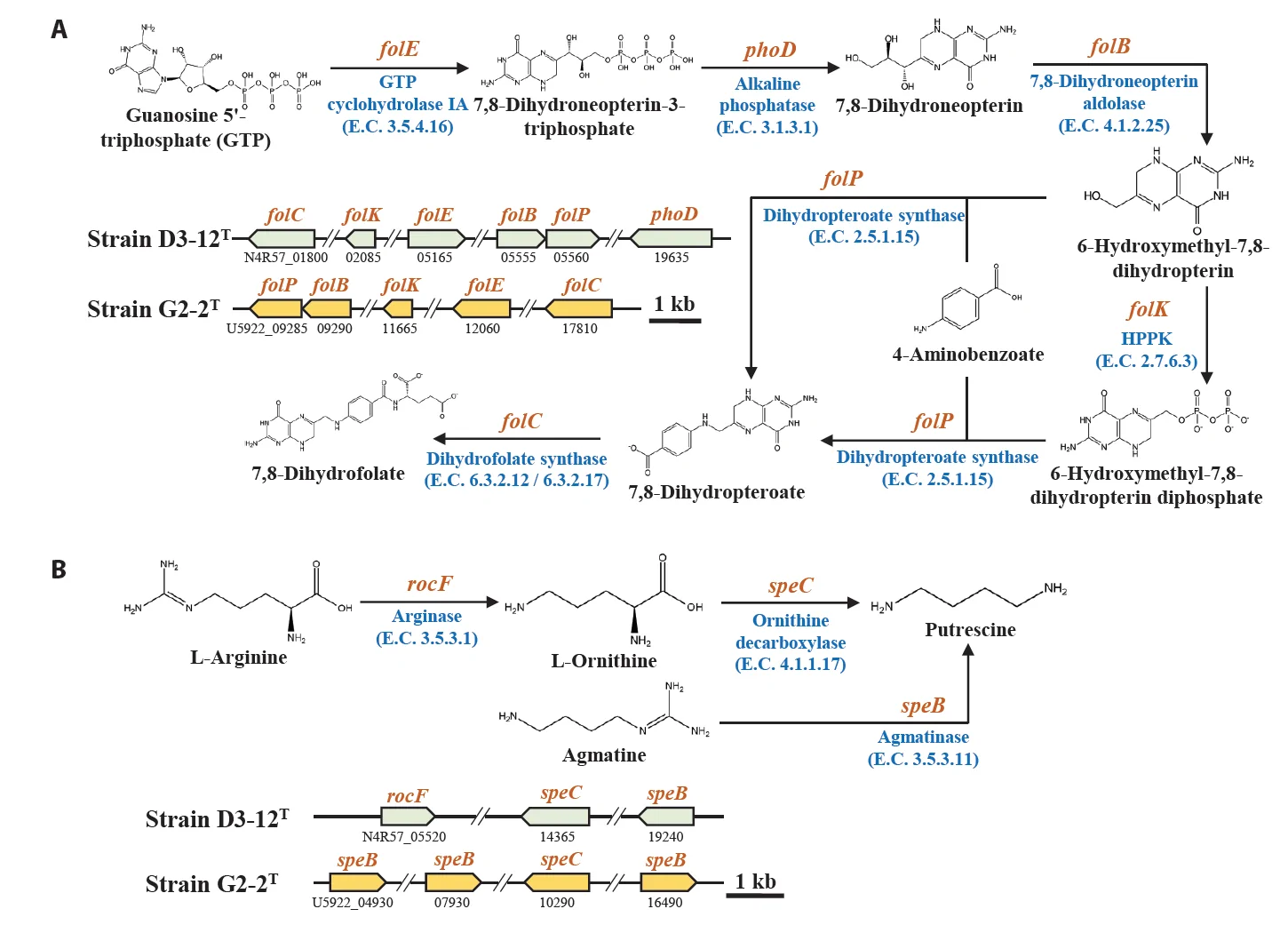
Biosynthetic pathways and associated genes for the biosynthesis of 7,8-dihydrofolate from GTP (A) and putrescine from L-arginine or agmatine (B) identified in strains D3-12^T^ and G2-2^T^. HPPK, 2-amino-4-hydroxy-6-hydroxymethyldihydropteridine diphosphokinase.

**Fig. 4.**
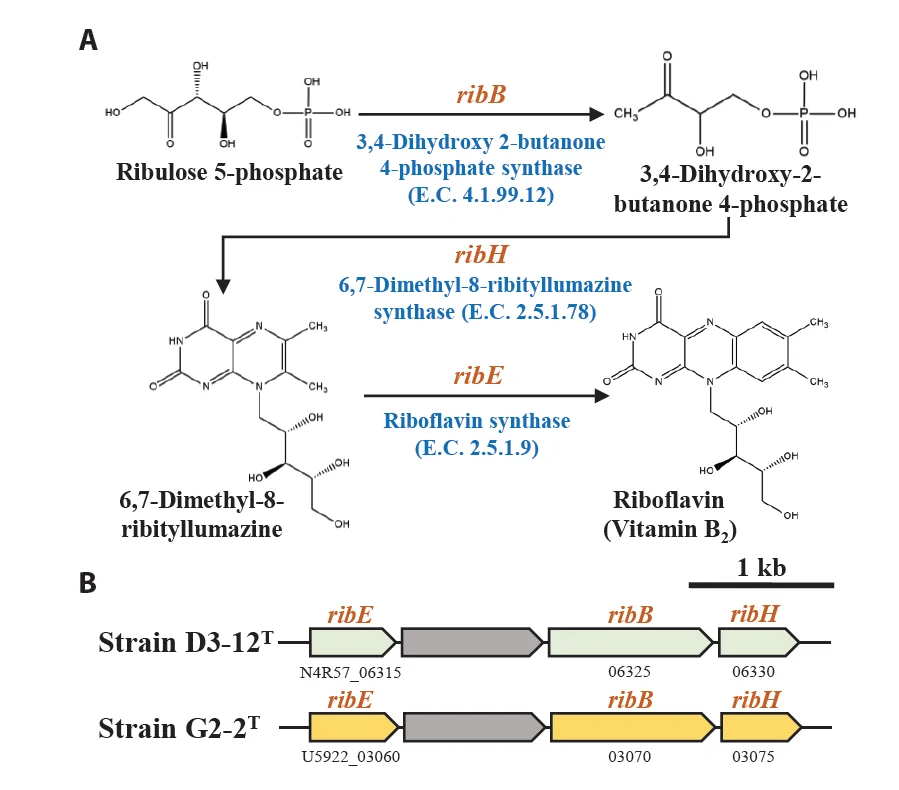
Metabolic pathway (A) and associated gene clusters (B) for the biosynthesis of the riboflavin from ribulose 5-phosphate identified in strains D3-12^T^ and G2-2^T^.

**Fig. 5.**
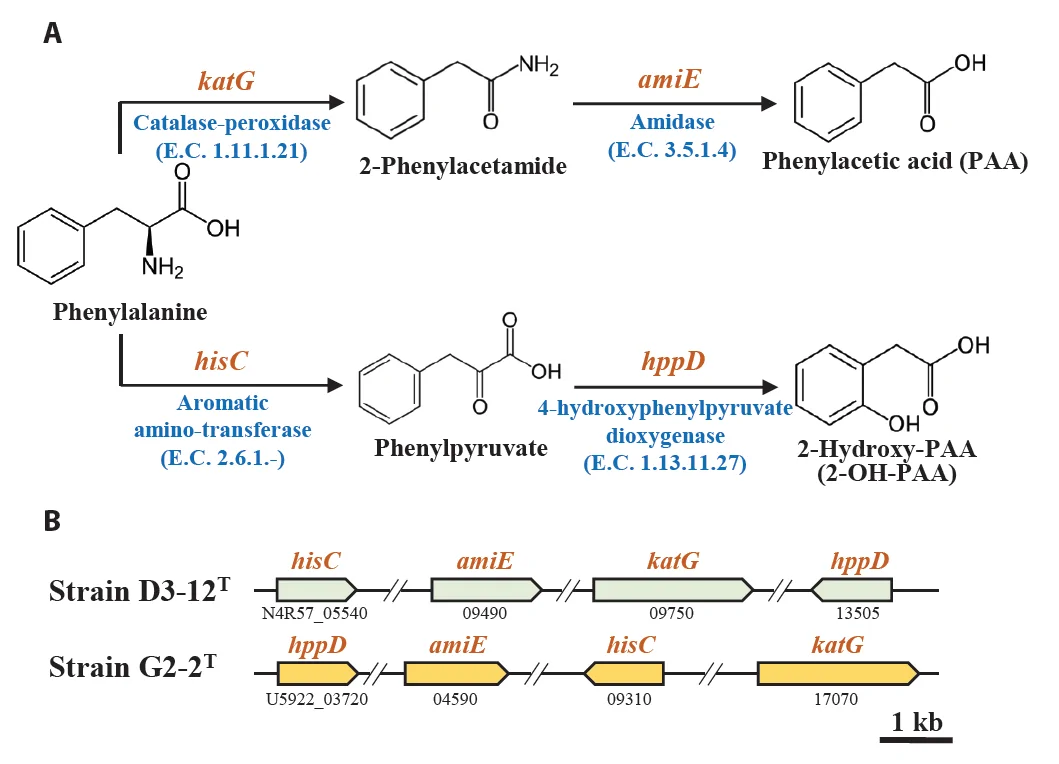
Metabolic pathway (A) and associated genes (B) for the biosynthesis of phenylacetic acid (PAA) and 2-hydroxy-PAA (2-OH-PAA) from phenylalanine identified in strains D3-12^T^ and G2-2^T^.

Marine bacteria in the phycosphere can influence their algal hosts through diverse metabolic interactions, including the production of vitamins, siderophores, compatible solutes, and nutrients (Kim et al., 2024b). Genomic analysis of strains D3-12ᵀ and G2-2ᵀ revealed the presence of multiple genes involved in the biosynthesis of compounds potentially beneficial to marine algal growth. Strain D3-12ᵀ possesses the complete set of genes (*folBCEKP* and *phoD*) required for the synthesis of dihydrofolate (DHF), a vitamin B9 derivative, from guanosine 5'-triphosphate (GTP). In contrast, strain G2-2ᵀ contains the *folBCEKP* genes but lacks *phoD*, which encodes alkaline phosphatase (Fig. 3A), suggesting that it may synthesize DHF from 7,8-dihydroneopterin rather than directly from GTP. Strain D3-12ᵀ also carries genes for putrescine production via two pathways: from arginine (*rocF* and *speC*) and from agmatine (*speB*) (Fig. 3B). Strain G2-2ᵀ, however, lacks *rocF* and appears capable of synthesizing putrescine only from agmatine. Additionally, only strain D3-12ᵀ harbors the *cbiBP* and *cobASU* genes involved in cobalamin (vitamin B_12_) biosynthesis from cobyrinate *a*,*c*-diamide, suggesting a potential role in supporting vitamin B_12_-dependent algal metabolism (Croft et al., 2005). Both strains possess the ribBEH gene cluster for riboflavin (vitamin B_2_) synthesis from ribulose-5-phosphate (Fig. 4). Furthermore, genes involved in the biosynthesis of phenylacetic acid (*katG* and *amiE*) and 2-hydroxy-phenylacetic acid (*hisC* and *hppD*) from L-phenylalanine were identified in both genomes (Fig. 5); these hormone-like compounds are known to promote algal growth and enhance stress tolerance (Kim et al., 2024b). Lastly, both strains encode bacterioferritin (*bfr*), a siderophore-related protein that facilitates iron acquisition and may further contribute to algal health. Collectively, these genomic features suggest that strains D3-12ᵀ and G2-2ᵀ possess metabolic traits that support symbiotic interactions with marine algae and potentially promote host growth within the phycosphere.

### Phenotypic and biochemical characteristics

Strains D3-12^T^ and G2-2^T^ exhibited robust growth on MA. Strain D3-12^T^ also grew well on NA supplemented with 2% NaCl, whereas strain G2-2^T^ showed slow growth. Both strains exhibited weak growth on R2A agar and TSA (with 2% NaCl), and only strain D3-12^T^ showed limited growth on LB agar with 2% NaCl. Cells of strains D3-12^T^ and G2-2^T^ were Gram-stain-negative, non-motile rods, measuring 1.1–1.2 µm wide and 2.1–2.2 µm long for strain D3-12ᵀ, and 1.0–1.1 µm wide and 2.2–2.3 µm long for strain G2-2ᵀ (Fig. S2). Neither strain exhibited anaerobic growth, confirming their strictly aerobic nature. Several phenotypic features—including aerobic metabolism, motility, catalase and oxidase activities, indole production, hydrolysis of gelatin, casein, and starch, and the assimilation of D-mannitol, capric acid, and phenylacetic acid—were consistent with characteristics of other *Roseobacteraceae* species (Table 2). However, strains D3-12ᵀ and G2-2ᵀ could be distinguished from closely related species by various traits, including glucose fermentation, arginine dihydrolase activity, and the assimilation of D-mannose, D-glucose, L-arabinose, and D-maltose.

### Chemotaxonomic characteristics

The sole respiratory isoprenoid quinone identified in both strains D3-12ᵀ and G2-2ᵀ was ubiquinone-10 (Q-10), consistent with the predominant quinone found in other members of the family *Roseobacteraceae* (Coe et al., 2023; Feng et al., 2018; Lee et al., 2020; Su et al., 2024; Thongphrom et al., 2017; Yang et al., 2018, 2023). Among the major fatty acids (> 5% of total), both strains contained summed feature 8 (comprising C_18:1_ *ω*7*c* and/or C_18:1_ *ω*6*c*) and C_16:0_ (Table S5). In addition, strain D3-12ᵀ possessed 11-methyl-C_18:1_ *ω*7*c* as a major component, which was absent in strain G2-2ᵀ, suggesting a chemotaxonomic distinction between the two strains. Although the overall fatty acid profiles of D3-12ᵀ and G2-2ᵀ were similar to those of closely related *Roseobacteraceae* species, notable differences were observed in the relative abundance of 1-methyl-C_18:1_ *ω*7*c*, C18:0, and cyclo-C19:0 ω8c (Table S5). The major polar lipids in strain D3-12ᵀ included PG, PE, and PC, along with an unidentified aminolipid and three unidentified lipids. In contrast, strain G2-2ᵀ contained PG and DPG, an unidentified aminolipid, and two unidentified lipids (Fig. S3). The presence or absence of PC, PE, and DPG between the two strains further supports their differentiation at the genus level. Nonetheless, both strains exhibited polar lipid profiles generally consistent with other *Roseobacteraceae* species (Table 2).

### Taxonomic conclusion

Based on phylogenetic analyses of 16S rRNA gene and whole-genome sequences, along with physiological and chemotaxonomic characteristics, strains D3-12ᵀ and G2-2ᵀ are proposed to represent two novel and distinct genera within the family *Roseobacteraceae*. Accordingly, we propose the names *Phycobium rhodophyticola* gen. nov., sp. nov. for strain D3-12ᵀ, and *Aliiphycobium algicola* gen. nov., sp. nov. for strain G2-2ᵀ.

### Description of *Phycobium* gen. nov.

*Phycobium* (Phy.co′bi.um. Gr. neut. n. *phykos*, seaweed; Gr. masc. n. *bios*, life; N.L. neut. n. *Phycobium*, a living form from an alga).

Cells are Gram-stain-negative, strictly aerobic, and non-motile rods. Oxidase and catalase activities are positive. Nitrate is not reduced to nitrite. Q-10 is identified as the sole respiratory quinone. The major cellular fatty acids (> 5%) are summed feature 8 (C_18:1_ *ω*7*c* and/or C_18:1_ *ω*6*c*), 11-methyl-C_18:1_ *ω*7*c*, and C_16:0_. The major polar lipids are PC, PG, and PE. Phylogenetically, the genus is a member of the family *Roseobacteraceae* within the order Rhodobacterales of the phylum *Pseudomonadota*. The type species is *Phycobium rhodophyticola*.

### Description of *Phycobium rhodophyticola* sp. nov.

*Phycobium rhodophyticola* (rho.do.phy.ti′co.la. N.L. neut. pl. n. Rhodophyta, the division of the red algae; L. suffix. -*cola* (from L. masc. or fem. n. *incola*), inhabitant, dweller; N.L. masc. n. *rhodophyticola*, inhabitant of *Rhodophyta*).

In addition to the characteristics described for the genus, this species exhibits the following traits. Colonies grown on MA are smooth and round. Growth occurs between 10–35°C (optimal at 30°C) and pH 6.0–9.0 (optimal at pH 7.0), with NaCl concentrations of 1.0–6.0% (w/v) (optimal at 2.0–3.0%). Esculin hydrolysis is positive, while hydrolysis of tyrosine, casein, starch, gelatin, Tween 20, and Tween 80 is negative. Fermentation of D-glucose is positive, but indole production is negative. Positive for arginine dihydrolase, urease, and *β*-galactosidase activities. Assimilates N-acetyl-glucosamine, trisodium citrate, and malic acid, but not D-glucose, L-arabinose, D-maltose, D-mannose, D-mannitol, potassium gluconate, adipic acid, capric acid, and phenylacetic acid.

The type strain is D3-12^T^ (= KACC 22712^T^ = JCM 35528^T^), isolated from the phycosphere of the marine red alga *Melanothamnus japonicus*, collected from a coastal region in Korea. The genome size of the strain is 4,496 kb, and its DNA G + C content is 59.9%, as determined from the whole genome sequence. The GenBank accession numbers for the 16S rRNA gene and the genome sequences of strain D3-12^T^ are ON077633 and CP104793, respectively.

### Description of *Aliiphycobium* gen. nov.

*Aliiphycobium* (A.li.i.phy.co′bi.um. L. masc. adj. *alius*, other, another; N.L. masc. n. *Phycobium*, a bacterial generic name; N.L. masc. n. *Aliiphycobium*, the other *Phycobium*).

Cells are Gram-stain-negative, strictly aerobic, and non-motile rods without flagella. Oxidase and catalase activities are positive. Nitrate is reduced to nitrite. Q-10 is identified as the sole respiratory quinone. The major cellular fatty acids are summed feature 8 (C_18:1_ *ω*7*c* and/or C_18:1_ *ω*6*c*) and C_16:0_. The major polar lipids are PG and DPG. Phylogenetically, the genus is a member of the family *Roseobacteraceae* within the order Rhodobacterales of the phylum *Pseudomonadota*. The type species is *Aliiphycobium algicola*.

### Description of *Aliiphycobium algicola* sp. nov.

*Aliiphycobium algicola*, (al.gi′co.la. L. fem. n. alga, an alga; L. suffix. -*cola* (from L. masc. or fem. n. *incola*), inhabitant, dweller; N.L. masc. or fem. n. *algicola*, an alga dweller).

In addition to the characteristics described for the genus, this species exhibits the following traits. Colonies grown on MA are circular and smooth. Growth occurs between 10–35°C (optimal at 30°C) and pH 6.0–8.0 (optimal at pH 7.0), with NaCl concentrations of 1.0–6.0% (w/v) (optimal at 2.0%). Hydrolysis of esculin, tyrosine, casein, starch, gelatin, Tween 20, and Tween 80 is negative. Fermentation of D-glucose is positive, but indole production is negative. Positive for arginine dihydrolase activity, but negative for urease and *β*-galactosidase activities. Assimilates adipic acid, but not D-glucose, L-arabinose, D-maltose, D-mannose, D-mannitol, N-acetyl-glucosamine, trisodium citrate, potassium gluconate, malic acid, capric acid and phenylacetic acid.

The type strain is G2-2^T^ (= KACC 22602^T^ = JCM 35752^T^), isolated from the phycosphere of the marine red alga *Chondrus* species, collected from a coastal region in Korea. The genome size of the strain is 3,786 kb, and its DNA G + C content is 60.2%, as determined from the whole genome sequence. The GenBank accession numbers for the 16S rRNA gene and the genome sequences of strain G2-2^T^ are OL985676 and CP170201– CP170203, respectively.

## Figures and Tables

**Table 1.** General genomic features^†^ of strains D3-12^T^ and G2-2^T^ and their closely related taxa of the family *Roseobacteraceae*. Taxa: 1, strain D3-12^T^ (CP104793); 2, strain G2-2^T^ (CP170201–3); 3, *Rhodalgimonas zhirmunskyi* 10Alg 79^T^ (JANFFA000000000); 4, *Ponticoccus litoralis* KCCM 90028^T^ (JBDNCH000000000); 5, *Aquicoccus porphyridii* L1 8-17^T^ (VINQ00000000); 6, *Marimonas lutisalis* GH1-19^T^ (SKCI00000000); 7, *Marimonas arenosa* KCTC 52189^T^ (JANHAX000000000); 8, *Cognatishimia maritima* DSM 28223^T^ (FQWM00000000)

Feature	1	2	3	4	5	6	7	8
Genome status (no. of contigs)^‡^	C (1)	C (3)	D (73)	D (5)	D (256)	D (14)	D (38)	D (14)
Genome size (kb)	4,496	3,786	3,755	4,790	4,514	4,320	4,381	3,285
G + C content (%)	59.9	60.2	62.1	67.3	63.2	63.2	63.3	56.3
No. of total genes	4,393	3,681	3,626	4,772	4,560	4,260	4,277	3,304
No. of protein-coding genes	4,049	3,393	3,548	3,931	4,379	4,151	4,186	3,214
No. of total RNA genes	48	48	48	59	52	50	50	65
No. of tRNA genes	42	42	41	47	43	44	44	50
No. of rRNA (16S, 23S, 5S) operons	1	1	1	3	2	1	1	4
No. noncoding RNA genes	3	3	3	3	3	3	3	3

† The bioinformatic analysis of the genomes was carried out using the NCBI prokaryotic genome annotation pipeline (www.ncbi.nlm.nih.gov/genome/annotation_prok/).

‡ C, complete; D, draft

**Table 2.** Differential phenotypic characteristics between strains D3-12^T^ and G2-2^T^ and their closely related taxa of the family *Roseobacteraceae*. Taxa: 1, strain D3-12^T^ (this study); 2, strain G2-2^T^; 3, *Rhodalgimonas zhirmunskyi* KCTC 72611^T^ (Nedashkovskaya et al., 2023); 4, *Ponticoccus litoralis* KCCM 90028^T^ (Hwang and Cho, 2008); 5, *Aquicoccus porphyridii* KACC 18806^T^ (Feng et al., 2018); 6, *Marimonas lutisalis* KCTC 62376^T^ (Lee et al., 2020); 7, *Marimonas arenosa* KCTC 52189^T^ (Thongphrom et al., 2017); 8, *Cognatishimia maritima* KCTC 23347^T^ (Park et al., 2012). All strains are positive for the following characteristics: activity^*^ of catalase and oxidase. All strains are negative for the following characteristics: Gram-staining, anaerobic growth, indole production^*^, hydrolysis^*^ of gelatin and assimilation* of capric acid. Symbols: +, positive; –, negative; w, weakly positive

Characteristic	1	2	3	4	5	6	7	8
Isolation source	Marine alga	Marine alga	Marine alga	Coastal seawater	Marine alga	Tidal mudflat	Sea sand	Seawater
Colony color	Beige	Beige	Beige	Cream	Beige^*^	Beige	Beige	Cream
Cell morphology	Rod	Rod	Rod	Coccus	Coccus	Rod	Rod	Rod
Cell motility	–	–	–	–	–	+	–	+
Range for growth:								
Temperature (°C)	10–35	10–35	4–40	10–37	20–40	10–40	20–37	10–37
pH	6.0–9.0	6.0–8.0	6.0–9.0	6.0–8.0	6.0–10.0	6.0–9.0	6.5–10.0	6.0–8.0
NaCl (%, w/v)	1–6	1–6	0–5	1–15	0–7	1–9	0–6	0–7
Nitrate reduction^*^	–	+	–	+	–	–	+	–
Glucose fermentation^*^	+	+	–	+	–	–	–	–
Hydrolysis^*^ of:								
Casein	–	–	–	–	–	–	–	+
Esculin	+	–	*w*	+	–	–	–	–
Starch	–	–	*w*	–	–	–	–	–
Tween 20, Tween 80	–	–	+	+	–	–	–	–
Tyrosine	–	–	–	+	+	–	–	+
Enzyme activity^*^ of:								
Arginine dihydrolase	+	+	–	+	–	–	–	–
Urease	+	–	–	+	–	–	–	–
*β*-Galactosidase	*w*	–	–	+	+	–	–	–
Assimilation^*^ of:								
D-Glucose	–	–	+	*w*	+	–	–	+
L-Arabinose, D-maltose	–	–	+	+	–	+	*w*	–
D-Mannose	–	–	+	*w*	+	+	*w*	+
D-Mannitol	–	–	+	–	–	–	–	+
*N*-Acetyl-glucosamine	*w*	–	+	–	–	–	–	–
Trisodium citrate	*w*	–	–	–	–	–	–	–
Potassium gluconate	–	–	+	+	–	–	+	–
Phenylacetic acid	–	–	+	–	–	–	–	–
Adipic acid	–	*w*	+	*w*	–	–	+	–
Malic acid	+	–	+	+	+	+	*w*	+
Major polar lipids^†^	PC, PG, PE	PG, DPG	PC, PG, PE	PC, PG, PE	PC, PG, PE	PC, PG, PE, DPG	PC, PG, PE	PC, PG, PE

* These analyses were conducted under the same conditions in this study.

† PC, phosphatidylcholine; PG, phosphatidylglycerol; PE, phosphatidylethanolamine; DPG, diphosphatidylglycerol

## Data Availability

The GenBank/EMBL/DDBJ accession numbers for the 16S rRNA gene sequences of strains D3-12^T^ and G2-2^T^ are ON077633 and OL985676, respectively, and those for the genome sequences of strain D3-12^T^, strain G2-2^T^, and *Ponticoccus litoralis* KCCM 90028^T^ are CP104793, CP170201–3, and JBDNCH000000000, respectively.

## Supplementary Information

Fig. S1. Maximum-likelihood (A) and maximum-parsimony (B) trees showing the phylogenetic relationships between strains D3-12^T^ and G2-2^T^ and their closely related taxa, based on 16S rRNA gene sequences. Only bootstrap values exceeding 70% are indicated on the nodes as percentages from 1000 replicates. *Thioclava pacifica* DSM 10166^T^ (KX618934) was used as an outgroup. The scale bars in panels A and B indicate nucleotide changes per nucleotide and throughout the entire sequence, respectively.

Fig. S2. Transmission electron micrographs of negatively stained (using 2% uranyl acetate) cells showing the general morphologies of strains D3-12^T^ (A) and G2-2^T^ (B) grown on marine agar at 30°C for 2 days.

Fig. S3. Two-dimensional thin-layer chromatograms (TLC) showing the polar lipids profiles of strains D3-12^T^ and G2-2^T^. Solvent systems: (I) chloroform-methanol-water (65:25:4, v/v/v) and (II) chloroform-acetic acid-methanol-water (80:15:12:4, v/v/v/v). The TLC plates were sprayed with 10% ethanolic molybdophosphoric acid (A), ninhydrin (B), Dittmer-Lester (C) and Dragendorff (D) reagents for the detection of total polar lipids, aminolipids, phospholipids, and phosphatidylcholine, respectively. The TLC plates for glycolipid detection using *α*-naphthol/sulfuric acid reagent were not displayed, as no spots were observed. Abbreviations: PC, phosphatidylcholine; PG, phosphatidylglycerol; PE, phosphatidylethanolamine; DPG, diphosphatidylglycerol; AL, unidentified aminolipid; L, unidentified lipid.

Table S1. Potential ecological distribution of strains D3-12^T^ and G2-2^T^ assessed by comparing their 16S rRNA gene sequences against metagenomic 16S rRNA amplicon datasets using the IMNGS platform, with a sequence similarity threshold of 99.0%. “Matched No.” indicates the number of metagenomic datasets including sequences matching the 16S rRNA gene of each strain, while “ARA” represents the average relative abundance of these sequences within the respective datasets. ARA values below 0.05% for both strains in all datasets were excluded.

Table S2. Average nucleotide identity (ANI) and digital DNA-DNA hybridization (dDDH) values among strains D3-12^T^ and G2-2^T^ and their closely related taxa of the family *Roseobacteraceae*

Table S3. Average amino acid identity (AAI) and percentage of conserved proteins (POCP) among strains D3-12^T^ and G2-2^T^ and their closely related taxa of the family *Roseobacteraceae*

Table S4. Profiles of carbohydrate-active enzyme (CAZyme) genes identified in the genomes of strains D3-12^T^ and G2-2^T^, along with those of their closely related type strains

Table S5. Cellular fatty acid compositions (%) of strains D3-12^T^ and G2-2^T^ and closely related taxa of the family *Roseobacteraceae*
