# Supplementary material for: Phycobium rhodophyticola gen. nov., sp. nov. and Aliiphycobium algicola gen. nov., sp. nov., isolated from the phycosphere of marine red algae
Source: J Microbiol. 2025 Jun 30;63(6):e2503014. doi: 10.71150/jm.2503014 (PMC13577095; doi:10.71150/jm.2503014)

Fig. S1. Maximum-likelihood (A) and maximum-parsimony (B) trees showing the phylogenetic relationships between strains D3-12^{T} and G2-2^{T} and their closely related taxa, based on 16S rRNA gene sequences. Only bootstrap values exceeding 70% are indicated on the nodes as percentages from 1000 replicates. *Thioclava pacifica* DSM 10166^{T} (KX618934) was used as an outgroup. The scale bars in panels A and B indicate nucleotide changes per nucleotide and throughout the entire sequence, respectively.

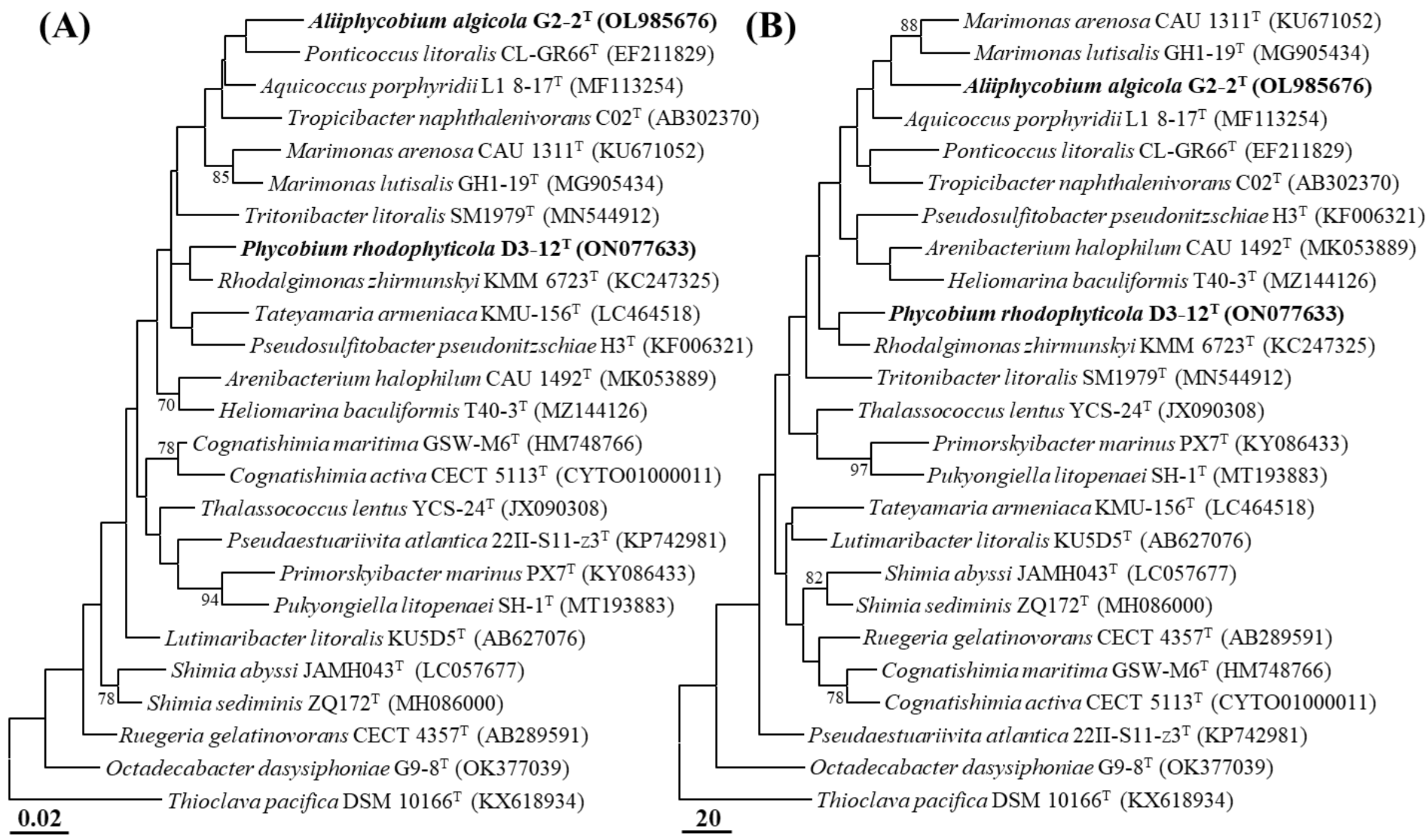

Supplement: Supplementary file 1 [file jm-2503014-Supplementary-Fig-S1.pdf]
