# Supplementary material for: Phycobium rhodophyticola gen. nov., sp. nov. and Aliiphycobium algicola gen. nov., sp. nov., isolated from the phycosphere of marine red algae
Source: J Microbiol. 2025 Jun 30;63(6):e2503014. doi: 10.71150/jm.2503014 (PMC13577095; doi:10.71150/jm.2503014)

Fig. S2. Transmission electron micrographs of negatively stained (using 2% uranyl acetate) cells showing the general morphologies of strains D3-12^T (A) and G2-2^T (B) grown on marine agar at 30°C for 2 days.

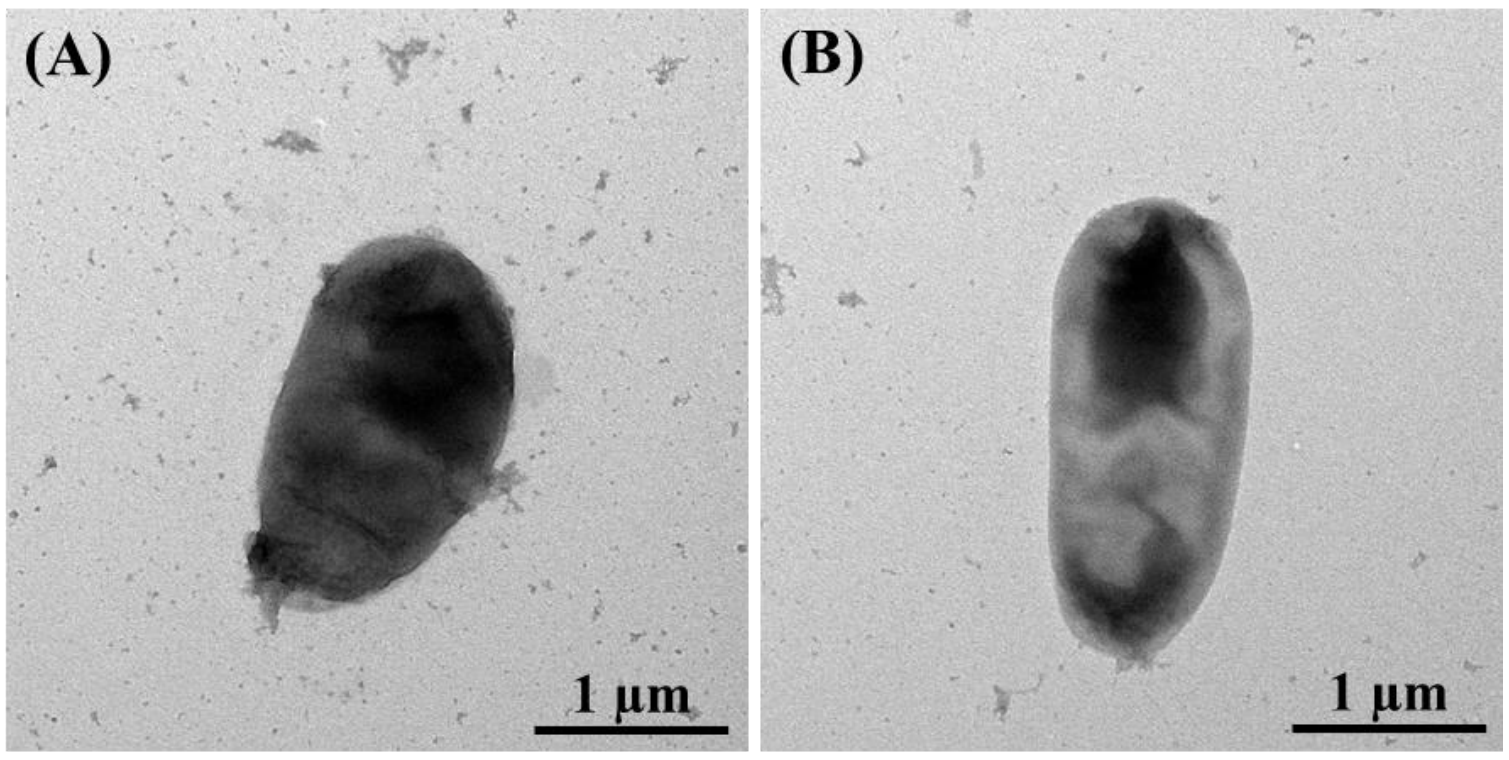

Supplement: Supplementary file 2 [file jm-2503014-Supplementary-Fig-S2.pdf]
