# Supplementary material for: Phycobium rhodophyticola gen. nov., sp. nov. and Aliiphycobium algicola gen. nov., sp. nov., isolated from the phycosphere of marine red algae
Source: J Microbiol. 2025 Jun 30;63(6):e2503014. doi: 10.71150/jm.2503014 (PMC13577095; doi:10.71150/jm.2503014)

Fig. S3. Two-dimensional thin-layer chromatograms (TLC) showing the polar lipids profiles of strains D3-12^{T} and G2-2^{T}. Solvent systems: (I) chloroform-methanol-water (65:25:4, v/v/v) and (II) chloroform-acetic acid-methanol-water (80:15:12:4, v/v/v/v). The TLC plates were sprayed with 10% ethanolic molybdophosphoric acid (A), ninhydrin (B), Dittmer-Lester (C) and Dragendorff (D) reagents for the detection of total polar lipids, aminolipids, phospholipids, and phosphatidylcholine, respectively. The TLC plates for glycolipid detection using *α*-naphthol/sulfuric acid reagent were not displayed, as no spots were observed. Abbreviations: PC, phosphatidylcholine; PG, phosphatidylglycerol; PE, phosphatidylethanolamine; DPG, diphosphatidylglycerol; AL, unidentified aminolipid; L, unidentified lipid.

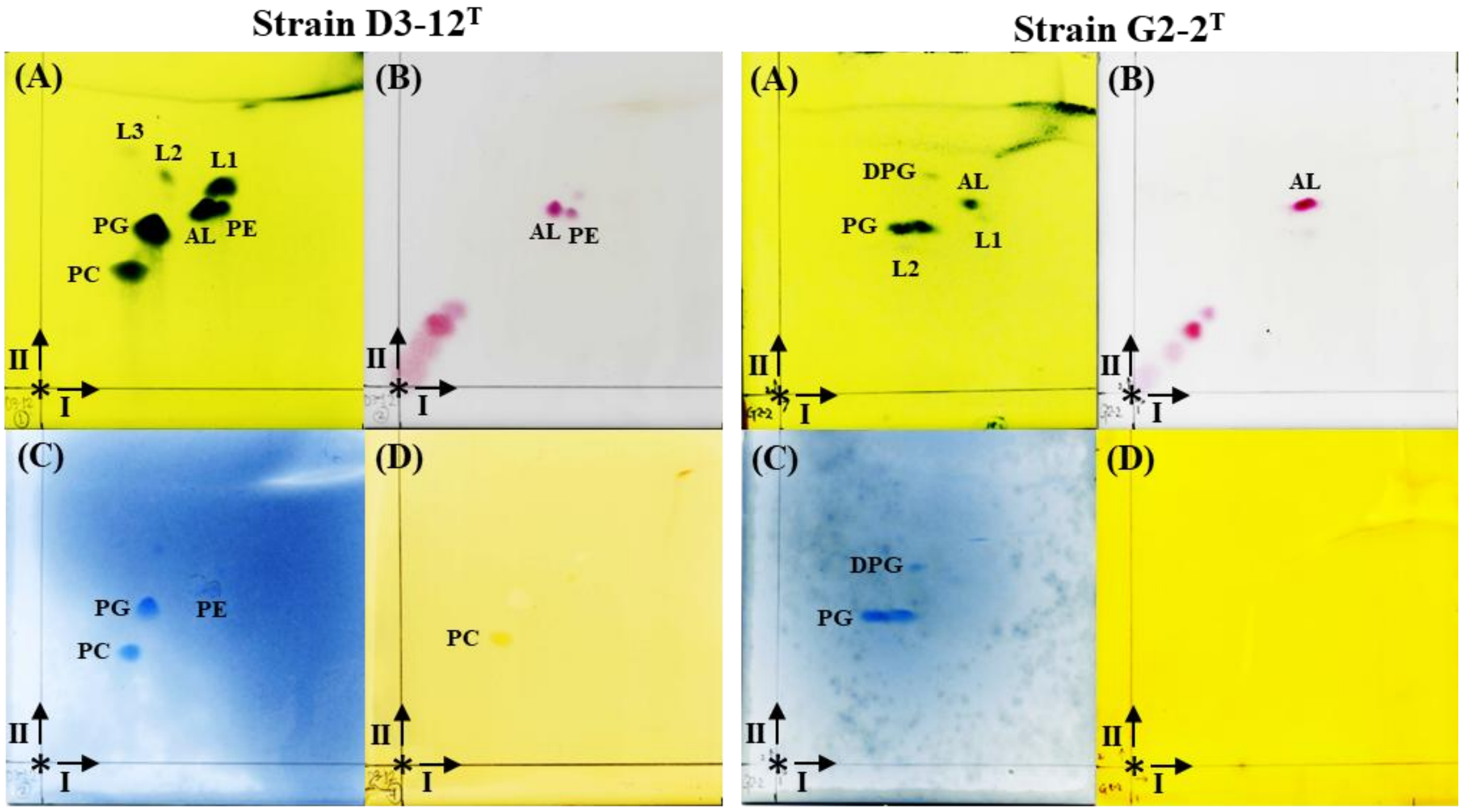

Supplement: Supplementary file 3 [file jm-2503014-Supplementary-Fig-S3.pdf]
