# Supplementary material for: Phycobium rhodophyticola gen. nov., sp. nov. and Aliiphycobium algicola gen. nov., sp. nov., isolated from the phycosphere of marine red algae
Source: J Microbiol. 2025 Jun 30;63(6):e2503014. doi: 10.71150/jm.2503014 (PMC13577095; doi:10.71150/jm.2503014)
Supplement: Supplementary file 4 [file jm-2503014-Supplementary-Table-S1.pdf]

Table S1. Potential ecological distribution of strains D3-12^T and G2-2^T assessed by comparing their 16S rRNA gene sequences against metagenomic 16S rRNA amplicon datasets using the IMNGS platform, with a sequence similarity threshold of 99.0%. "Matched No." indicates the number of metagenomic datasets including sequences matching the 16S rRNA gene of each strain, while "ARA" represents the average relative abundance of these sequences within the respective datasets. ARA values below 0.05% for both strains in all datasets were excluded.

Metagenome datasets	No. of datasets	Strain D3-12^T			Strain G2-2^T		
		Matched no.	Prevalence (%)	ARA (%)	Matched no.	Prevalence (%)	ARA (%)
Panulirus ornatus	20	1	5.00	5.06	0	0	0.00
Terrestrial metagenome	887	1	0.11	5.01	0	0	0.00
Marine metagenome	37438	108	0.29	1.56	3	0.01	0.01
Seawater metagenome	3225	4	0.12	0.11	0	0	0.00
Seminavis robusta	9	6	66.67	0.69	5	55.56	0.18
Algae metagenome	689	3	0.44	0.06	1	0.15	0.01
Marine sediment metagenome	3244	3	0.09	0.01	1	0.03	0.41
Sponge metagenome	653	0	0	0.00	1	0.15	0.09
Algae metagenome	689	3	0.44	0.06	1	0.15	0.01
Compost metagenome	383	1	0.26	0.01	6	1.57	0.42
Plant metagenome	12101	41	0.34	0.24	1	0.01	0.18
Insect metagenome	1544	0	0	0.00	1	0.06	1.00
Human blood metagenome	84	0	0	0.00	2	2.38	0.41
Fish metagenome	706	0	0	0.00	17	2.41	0.25
Human metagenome	19843	0	0	0.00	2	0.01	0.10
Freshwater metagenome	14593	2	0.01	0.003	11	0.08	0.07
