# Supplementary material for: Phycobium rhodophyticola gen. nov., sp. nov. and Aliiphycobium algicola gen. nov., sp. nov., isolated from the phycosphere of marine red algae
Source: J Microbiol. 2025 Jun 30;63(6):e2503014. doi: 10.71150/jm.2503014 (PMC13577095; doi:10.71150/jm.2503014)
Supplement: Supplementary file 5 [file jm-2503014-Supplementary-Table-S2.pdf]

Table S2. Average nucleotide identity (ANI) and digital DNA-DNA hybridization (dDDH) values among strains D3-12^T and G2-2^T and their closely related taxa of the family *Roseobacteraceae*

Taxa: 1, strain D3-12^T (CP104793); 2, strain G2-2^T (CP170201–3); 3, *Rhodalgimonas zhirmunskyi* 10Alg 79^T (JANFFA000000000); 4, *Ponticoccus litoralis* KCCM 90028^T (JBDNCH000000000); 5, *Aquicoccus porphyridii* L1 8-17^T (VINQ00000000); 6, *Marimonas lutisalis* GH1-19^T (SKCI00000000); 7, *Marimonas arenosa* KCTC 52189^T (JANHAX000000000); 8, *Cognatishimia maritima* DSM 28223^T (FQWM00000000)

		ANI value (%)							
		**1**	**2**	**3**	**4**	**5**	**6**	**7**	**8**
dDDH value (%)	**1**	–	74.5	74.7	71.7	74.8	75.5	75.4	70.7
	**2**	18.7	–	74.5	71.4	75.0	75.4	75.4	70.0
	**3**	19.5	19.0	–	72.8	75.6	76.4	75.7	70.6
	**4**	19.0	19.4	19.8	–	73.0	73.7	73.0	70.5
	**5**	19.0	19.1	19.8	19.3	–	79.3	76.5	70.4
	**6**	19.8	19.1	20.3	19.5	21.8	–	78.2	70.9
	**7**	19.6	19.2	19.4	19.4	20.2	21.6	–	70.4
	**8**	19.5	21.0	19.7	20.3	20.5	19.5	19.4	–
