# Supplementary material for: Phycobium rhodophyticola gen. nov., sp. nov. and Aliiphycobium algicola gen. nov., sp. nov., isolated from the phycosphere of marine red algae
Source: J Microbiol. 2025 Jun 30;63(6):e2503014. doi: 10.71150/jm.2503014 (PMC13577095; doi:10.71150/jm.2503014)
Supplement: Supplementary file 6 [file jm-2503014-Supplementary-Table-S3.pdf]

Table S3. Average amino acid identity (AAI) and percentage of conserved proteins (POCP) among strains D3-12^T and G2-2^T and their closely related taxa of the family *Roseobacteraceae*

Taxa: 1, strain D3-12^T (CP104793); 2, strain G2-2^T (CP170201–3); 3, *Rhodalgimonas zhirmunskyi* 10Alg 79^T (JANFFA000000000); 4, *Ponticoccus litoralis* KCCM 90028^T (JBDNCH000000000); 5, *Aquicoccus porphyridii* L1 8-17^T (VINQ00000000); 6, *Marimonas lutisalis* GH1-19^T (SKCI00000000); 7, *Marimonas arenosa* KCTC 52189^T (JANHAX000000000); 8, *Cognatishimia maritima* DSM 28223^T (FQWM00000000)

		POCP value (%)							
		**1**	**2**	**3**	**4**	**5**	**6**	**7**	**8**
AAI value (%)	**1**	–	61.3	68.3	45.6	62.0	65.2	64.6	51.3
	**2**	75.0	–	66.2	44.5	62.9	62.4	62.5	52.4
	**3**	74.1	74.4	–	50.3	68.6	70.5	70.4	57.0
	**4**	67.5	67.6	67.6	–	46.8	46.5	46.6	46.9
	**5**	74.4	74.7	74.1	67.8	–	66.6	65.2	53.9
	**6**	75.9	75.9	75.5	68.6	80.3	–	77.7	53.4
	**7**	76.6	76.6	75.1	67.9	76.1	78.5	–	53.7
	**8**	69.1	68.4	68.9	67.5	68.7	69.4	68.8	–
