# Supplementary material for: Phycobium rhodophyticola gen. nov., sp. nov. and Aliiphycobium algicola gen. nov., sp. nov., isolated from the phycosphere of marine red algae
Source: J Microbiol. 2025 Jun 30;63(6):e2503014. doi: 10.71150/jm.2503014 (PMC13577095; doi:10.71150/jm.2503014)
Supplement: Supplementary file 7 [file jm-2503014-Supplementary-Table-S4.pdf]

Table S4. Profiles of carbohydrate-active enzyme (CAZyme) genes identified in the genomes of strains D3-12^T and G2-2^T, along with those of their closely related type strains

Taxa: 1, strain D3-12^T (CP104793); 2, strain G2-2^T (CP170201–3); 3, *Rhodalgimonas zhirmunskyi* 10Alg 79^T (JANFFA000000000); 4, *Ponticoccus litoralis* KCCM 90028^T (JBDNCH000000000); 5, *Aquicoccus porphyridii* L1 8-17^T (VINQ00000000); 6, *Marimonas lutisalis* GH1-19^T (SKCI00000000); 7, *Marimonas arenosa* KCTC 52189^T (JANHAX000000000); 8, *Cognatishimia maritima* DSM 28223^T (FQWM00000000).

CAZyme categories	Taxa							
	**1**	**2**	**3**	**4**	**5**	**6**	**7**	**8**
Glycoside hydrolases (GHs)	10	7	10	15	10	11	11	12
GH1	0	0	0	0	0	0	0	1
GH3	1	1	1	1	1	1	1	1
GH5_37	0	0	0	0	0	0	1	0
GH8	0	0	0	1	0	0	0	0
GH13_18	1	0	1	1	1	0	0	0
GH13_23	0	0	0	1	0	0	0	1
GH23	4	1	3	4	2	5	2	1
GH26	0	0	0	0	0	0	1	0
GH36	0	0	0	0	0	0	0	1
GH42	0	0	0	0	0	0	0	2
GH63	0	0	0	0	0	0	0	1
GH77	0	0	0	1	0	0	0	0
GH102	1	1	1	1	1	1	1	1
GH103	3	4	4	4	4	4	4	2
GH108	0	0	0	1	0	0	0	0
GH128	0	0	0	0	1	0	1	0
GH154	0	0	0	0	0	0	0	1
Glycosyltransferases (GTs)	37	21	30	25	33	29	32	24
GT1	2	0	0	0	0	0	0	0
GT2	12	7	9	9	11	8	12	8
GT4	8	4	6	4	7	9	6	6
GT8	0	0	0	1	0	0	0	0
GT14	2	1	2	1	2	2	2	2
GT19	1	1	1	0	1	1	1	1
GT20	0	0	0	1	0	0	0	0
GT26	2	0	1	2	0	0	0	0
GT28	1	1	1	1	1	1	1	1
GT30	3	3	3	0	3	3	3	2
GT51	3	2	4	3	5	3	4	2
GT81	1	0	1	1	1	0	0	0
GT107	2	2	2	2	2	2	2	2
GT116	0	0	0	0	0	0	1	0
Carbohydrate esterases (CEs)	2	0	2	4	2	1	2	1
CE4	1	0	1	1	1	0	1	0
CE9	0	0	0	2	0	0	0	0
CE11	1	0	1	1	1	1	1	1
Auxiliary activities (AAs)	3	3	4	2	4	6	2	2
AA2	1	0	0	0	0	1	0	0
AA3_2	2	3	4	2	4	5	2	2
Total	52	31	46	46	49	47	47	39
