# Supplementary material for: Phycobium rhodophyticola gen. nov., sp. nov. and Aliiphycobium algicola gen. nov., sp. nov., isolated from the phycosphere of marine red algae
Source: J Microbiol. 2025 Jun 30;63(6):e2503014. doi: 10.71150/jm.2503014 (PMC13577095; doi:10.71150/jm.2503014)
Supplement: Supplementary file 8 [file jm-2503014-Supplementary-Table-S5.pdf]

Table S5. Cellular fatty acid compositions (%) of strains D3-12^T and G2-2^T and closely related taxa of the family *Roseobacteraceae*

Taxa: 1, strain D3-12^T; 2, strain G2-2^T; 3, *Rhodalgimonas zhirmunskyi* KCTC 72611^T; 4, *Ponticoccus litoralis* KCCM 90028^T; 5, *Aquicoccus porphyridii* KACC 18806^T; 6, *Marimonas lutisalis* KCTC 62376^T; 7, *Marimonas arenosa* KCTC 52189^T; 8, *Cognatishimia maritima* KCTC 23347^T. All data were obtained from this study. Data are presented as percentages of total fatty acids, with fatty acids accounting for less than 0.5% in all strains excluded from the results. Major components (> 5.0%) are highlighted in bold. tr, trace amount (< 0.5%); –, not detected.

Fatty acid	1	2	3	4	5	6	7	8
Saturated:								
$C_{10:0}$	0.6	0.6	0.7	0.8	tr	0.5	tr	tr
$C_{12:0}$	0.8	0.8	0.7	1.4	tr	1.2	tr	2.0
$C_{16:0}$	**5.9**	**17.8**	4.4	**8.1**	**6.9**	2.8	**12.3**	3.0
$C_{17:0}$	tr	1.4	1.2	tr	1.0	tr	1.8	1.0
$C_{18:0}$	2.3	1.2	0.8	**6.1**	1.7	3.4	4.2	3.3
Unsaturated:								
iso-$C_{17:1}$ $\omega 5c$	–	tr	–	0.6	–	–	–	0.5
11-methyl-$C_{18:1}$ $\omega 7c$	**10.1**	–	–	–	2.1	4.9	–	–
cyclo-$C_{19:0}$ $\omega 8c$	–	1.6	–	–	**10.8**	1.1	0.7	–
$C_{20:1}$ $\omega 7c$	tr	–	–	–	tr	0.7	–	–
Branched:								
iso-$C_{10:0}$	–	tr	–	0.7	–	–	–	0.7
iso-$C_{18:0}$	1.3	–	–	–	–	–	–	–
iso-$C_{19:0}$	–	tr	–	tr	–	–	–	0.5
Hydroxy:								
$C_{10:0}$ 3-OH	tr	tr	tr	–	tr	0.6	4.4	tr
$C_{11:0}$ 3-OH	–	–	0.7	–	–	–	–	–
$C_{12:0}$ 3-OH	2.1	2.2	2.2	0.9	0.7	0.8	tr	3.5
iso-$C_{12:0}$ 3-OH	–	0.6	tr	1.9	–	–	–	3.3
$C_{12:1}$ 3-OH	tr	tr	–	3.3	1.8	1.6	1.2	–
iso-$C_{17:0}$ 3-OH	–	–	0.6	–	–	–	–	–
Summed features*:								
3	tr	3.3	0.5	0.7	0.6	tr	0.7	0.7
7	–	–	tr	–	–	–	–	0.5
8	**75.1**	**67.6**	**85.6**	**73.9**	**69.9**	**78.2**	**72.7**	**78.5**

*Summed features are groups of fatty acids that cannot be resolved reliably using the chromatographic conditions chosen. The MIDI system groups these fatty acids together as one feature with a single percentage of the total. Summed features 3, 7, and 8 comprise $C_{16:1}$ $\omega 7c$ and/or $C_{16:1}$ $\omega 6c$, $C_{19:1}$ $\omega 7c$ and/or $C_{19:1}$ $\omega 6c$, and $C_{18:1}$ $\omega 7c$ and/or $C_{18:1}$ $\omega 6c$, respectively.
